# Impact of Lavender on Pain and Anxiety Levels Associated With Spine Procedures

**DOI:** 10.31486/toj.21.0013

**Published:** 2021

**Authors:** Maria Grabnar, Mary Joan Roach, Alaa Abd-Elsayed, Chong Kim

**Affiliations:** ^1^ Department of Physical Medicine and Rehabilitation, Case Western Reserve University/MetroHealth, Cleveland, OH; ^2^ Department of Anesthesiology, University of Wisconsin School of Medicine and Public Health, Madison, WI

**Keywords:** *Anti-anxiety agents*, *aromatherapy*, *lavandula*, *pain management*

## Abstract

**Background:** To reduce pain and anxiety associated with interventional pain procedures, sedation is often used, with benzodiazepines, opioids, and propofol the most commonly used classes of drugs for sedation. However, patient coherence and ability to communicate procedural pain and abnormal sensations help prevent adverse outcomes. Therefore, discovering alternative therapies to mitigate the anxiety and pain associated with these procedures and to minimize risk is important. The aim of our study was to investigate whether lavender has an effect on pain and anxiety associated with lumbar epidural steroid injections and lumbar medial branch blocks.

**Methods:** In this randomized controlled study, 54 subjects were randomly assigned to 1 of 3 intervention groups, and 46 patients were included in the final analysis: experimental lavender group (n=17), control almond oil group (n=15), and placebo sterile water group (n=14). Patients wore a mask infused with either lavender, almond oil, or water for 5 minutes prior to and during their procedure. Patients rated their anxiety using the State-Trait Anxiety Inventory prior to and after the procedure based on how they felt during the procedure. Patients rated their pain according to the numerical rating scale. Outcome measures were a comparison of pain among the 3 groups and a comparison of the change in anxiety before and after the procedure among the 3 groups.

**Results:** The lavender group demonstrated the highest mean change in anxiety scores (9.9) compared to almond oil (5.3) and water (3.6) preprocedurally vs postprocedurally. The lavender group also reported the lowest mean pain level (3.8) compared to almond oil (5.6) and water (5.6). However, none of the differences between groups showed statistical significance at the *P*<0.05 level.

**Conclusion:** Lavender may have a clinically beneficial effect on anxiety levels and pain reduction.

## INTRODUCTION

Back pain is one of the most common forms of musculoskeletal pain causing patients to seek medical care^1,2^ and is the leading cause of activity limitation and work loss throughout most of the world.^3^ Low back pain has a lifetime prevalence between 49% and 90%; therefore, most individuals will experience this condition at some point.^4^ Back pain secondary to spinal disease can be a challenging clinical condition to treat and can have several different etiologies. Based on evidence-based clinical practice guidelines for interventional techniques,^5^ the evidence for diagnostic lumbar facet joint nerve blocks is good, with 75% to 100% pain relief as the criterion standard. The same guidelines found good evidence for lumbar epidurals in managing disc herniation or radiculitis; fair evidence for managing axial or discogenic pain without disc herniation, radiculitis, or facet joint pain; and fair evidence for treating postsurgery syndrome with caudal epidural injections.

Interventional spine procedures such as lumbar epidural steroid injections and nerve blocks can be particularly painful or anxiety-provoking for patients. Introduction of needles can cause patient discomfort because of the presence of nociceptors in skin and underlying tissue.^6^ Studies have shown a correlation between pain and anxiety.^7-9^ To reduce pain and anxiety associated with interventional pain procedures, sedation is often used. Based on a 2017 survey that involved 337 pain physicians, sedation is used in approximately 50% of epidural injections and medial branch blocks.^10^ The survey also found that the most commonly used classes of drugs for sedation were benzodiazepines (97% of respondents), opioids (77% of respondents), and propofol (36% of respondents). Today's medical culture places an emphasis on patient satisfaction, which may be a reason that sedation is frequently used. However, studies have shown a lack of significant improvement in patient satisfaction with the use of sedation.^11^ The use of anxiolytics and analgesics carries a significant amount of risk involving erroneous nerve ablation and local anesthetic systemic toxicity; therefore, patient alertness during procedures involving spinal nerves is imperative. Patient coherence and ability to communicate procedural pain and abnormal sensations help prevent adverse outcomes.^12^ The American Society of Regional Anesthesia and Pain Medicine convened a group of experts to develop the Practice Advisory on Neurologic Complications in Regional Anesthesia and Pain Medicine, and per the guidelines, general anesthesia or heavy sedation removes the opportunity for the patient and/or physician to recognize, report, and respond to an atypical symptom during the interventional spine procedure, and the guidelines therefore recommend against this practice.^12,13^ In addition to impairing patient alertness during procedures, short-acting anxiolytic agents such as benzodiazepines and pain-relieving opiates are associated with side effects such as respiratory depression^14,15^ and may skew medial branch block outcomes. Benzodiazepines produce skeletal muscle relaxation and amnesia that may result in a false-positive response by alleviating baseline back pain^16^ or in a false-negative response by reducing pain tolerance.^17^ Therefore, discovering alternative therapies to mitigate the anxiety and pain associated with these procedures and to minimize risk and false outcomes is important.

Patients often use aromatherapy for pain relief, psychological comfort, and disease prevention, although evidence for the therapeutic efficacy of aromatherapy remains limited.^18^ Aromatherapy is defined as the therapeutic use of essential oils derived from plants and involves transmission of signals from the olfactory system to the brain, which regulates anxiety, depression, and mood disorders by secreting neurotransmitters such as serotonin and dopamine.^19^ While more than 40 plant derivatives have been identified for therapeutic use, lavender, eucalyptus, rosemary, chamomile, and peppermint are the most frequently used extracts.^20^

Lavender extracts are widely used in aromatherapy. Lavender aromatherapy has been reported to possess several therapeutic effects including antianxiety, mood stabilization, and analgesia.^21^ Lavender essential oil is primarily sold as an over-the-counter herbal medicine and has been granted the status of “Generally Recognized as Safe” by the US Food and Drug Administration, an indication of its safety when used as a dietary supplement.^22^

Clinically, lavender has been shown to decrease anxiety in patients scheduled to undergo colorectal surgery^23^ and has been shown to decrease anxiety associated with dental procedures.^24^ Lavender has also been shown to decrease pain during procedures, particularly when inhaled prior to a generally painful procedure such as catheter or needle insertion and chest tube removal.^25-27^ While the effects of lavender on various medical procedures have been investigated, no studies to the authors’ knowledge have examined the effects of lavender on fluoroscopic spine procedures. This study investigated whether inhalation of lavender essential oil reduced the pain and anxiety associated with interventional spine procedures, particularly lumbar epidural injections and medial branch blocks.

## METHODS

This study was approved by the MetroHealth Institutional Review Board. In addition, this study was registered at clinicaltrials.gov (NCT04257019). Clinical consent for the procedure was first obtained and then the subjects were consented for the study. After informed written consent was obtained, 56 patients ≥21 years who presented for lumbar epidural or lumbar medial branch block were included in the study. Exclusion criteria included an allergy or sensitivity to lavender or almond oil, a preexisting problem with the sense of smell, the inability to follow basic instructions relating to the design of the experiment, or the inability to answer questions regarding their pain or anxiety. Subjects were assigned to each group by a computer-generated randomizer.

The method of inhalation involved instillation of either lavender (100% *Lavandula x intermedia* [PipingRock Health Products]), sweet almond oil (100% *Prunus amygdalus dulcis* [PipingRock Health Products]), or sterile water into a standard surgical mask. Five drops were applied to different areas on the interior of the mask prior to asking the subject to don the mask on the day of the scheduled procedure.

After consent was obtained, basic demographic data were collected by medical record review and brief interview with the patient, including the procedure type, whether the patient took an anxiolytic or analgesic on that day, and whether the patient had had a lumbar epidural injection or medial branch block performed during the preceding 2 years. Patients were then asked to complete the State-Trait Anxiety Inventory (STAI) questionnaire regarding their current anxiety. The STAI is a psychological inventory based on a 4-point Likert scale and consists of 20 questions with total scores ranging from 20 to 80; higher scores indicate higher anxiety.

Patients were then taken to a quiet area away from the triage room, asked to put on a standard surgical mask, and instructed to breathe slowly and deeply for 5 minutes. Patients were then taken to the fluoroscopy suite for their scheduled procedure. Patients were told that they could remove their mask and terminate participation in the study at any point without any effect on the procedure. Immediately after completion of the procedure, patients were asked to remove the mask, taken back to the triage area, and asked to complete the STAI questionnaire again based on how they felt during the procedure. In addition, subjects were asked to rate their pain on the numerical rating scale (NRS) based on the pain they experienced during the procedure. The scale ranges from 0 to 10, with 0 being least severe and 10 being most severe.

The authors intended to recruit 75 subjects to have sufficient power, but the study had to be stopped because of critical mask shortages during the coronavirus disease 2019 (COVID-19) pandemic.

As stated earlier, 56 subjects were initially recruited, but 2 withdrew from the study. One subject in the lavender group reported that when filling out the STAI questionnaire, she became upset as it made her reflect on personal life events, and she requested to be withdrawn from the study. Another subject reported that she felt claustrophobic with the surgical mask on and also requested to be withdrawn from the study.

For the statistical analysis, only patients who answered all the questions were included, which was 46 of the 54 patients. Of those 46 patients, 6 took an anxiolytic (benzodiazepine-alprazolam, clonazepam, or diazepam) on the day of the procedure; none reported taking an analgesic. Patients were told that benzodiazepines, opioids, or nonsteroidal anti-inflammatory drugs were included in these categories. We did not ask patients to report scheduled daily medications such as selective serotonin reuptake inhibitors and anticonvulsants.

The 46 patients were randomized into the following treatment groups: 17 in the lavender group, 15 in the almond oil group, and 14 in the water group.

Outcome measures were comparison of pain among the 3 groups during the procedure and comparison of change in anxiety among the 3 groups before and after the procedure using analysis of variance. Statistical analysis was performed using SPSS software, version 26 (IBM Corp).

## RESULTS

Table 1 shows descriptive characteristics among groups. Mean age was similar among groups. All 3 groups had a higher percentage of females compared to males, but the differences were not statistically significant.

**Table 1.** Descriptive Characteristics by Treatment Group

	Treatment Group		
Variable	Lavender, n=17	Almond Oil, n=15	Water, n=14
Sex			
Male	8 (47.0)	5 (33.3)	4 (29.6)
Female	9 (53.0)	10 (66.7)	10 (71.4)
Mean age, years	54.2	57.1	52.6
Prior procedure	13 (76.5)	6 (40.0)	5 (35.7)
Anxiolytic or analgesic prior to the procedure	2 (11.8)	–	4 (28.6)

Note: Values are n (%) unless otherwise indicated.

**Figure 1.**
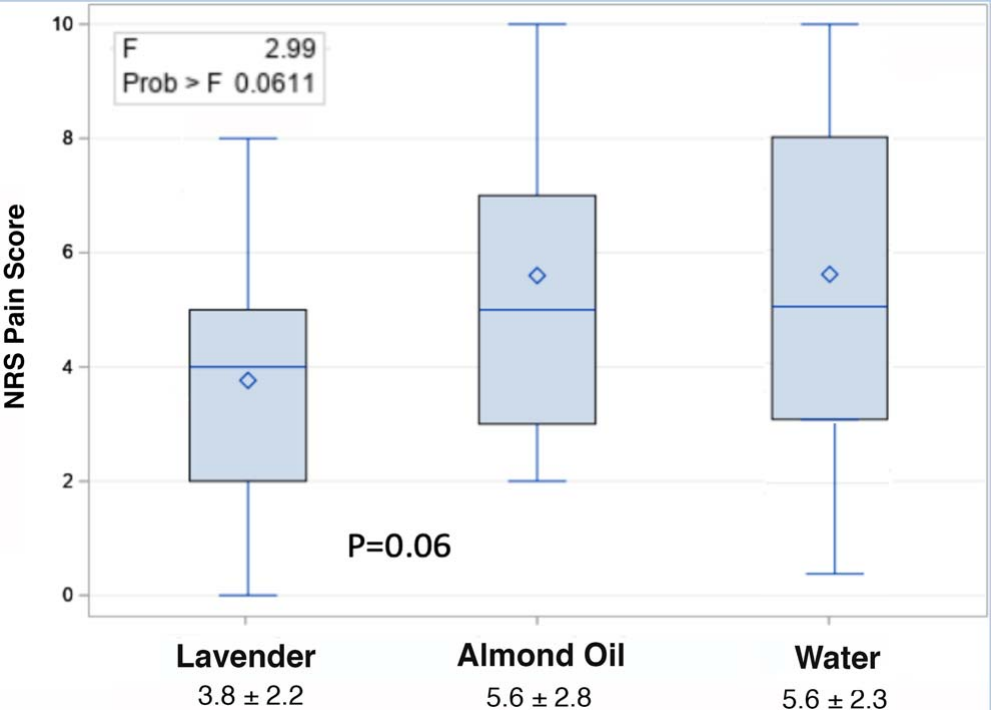
**Pain scores during procedures by treatment group.** NRS, numerical rating scale.

Figure 1 shows the pain level during the procedure among groups. Subjects in the lavender group showed a lower mean pain score (3.8) during the procedure than subjects in both the almond oil (5.6) and water (5.6) groups. Table 2 shows the between-group differences for pain levels. The between-group difference for lavender compared to both water and almond oil was –1.8, and the 95% CI did not cross 0.

**Table 2.** Differences in Pain Scores Between Treatment Groups During Procedures

Comparison	Difference Between Groups	95% CI
Lavender vs water	–1.8	–3.6 to –0.03
Lavender vs almond oil	–1.8	–3.6 to –0.09
Almond oil vs water	–0.3	–1.8 to –1.9

Note: Mean pain scores given in Figure 1 are rounded, resulting in the same pain score (5.6) for the almond oil and water groups. The difference between these groups, however, was calculated based on the full average pain score, and the difference between the two groups was –0.3 as shown in Table 2.

**Figure 2.**
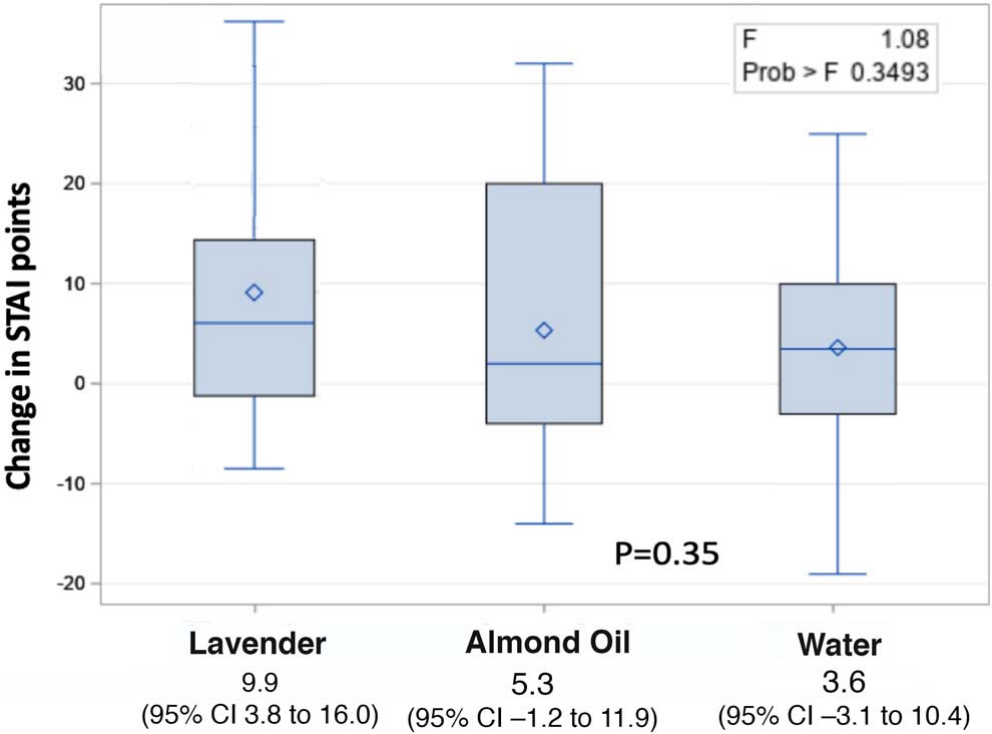
**Change in anxiety scores preprocedure vs postprocdure by treatment group.** STAI, State-Trait Anxiety Inventory.

Figure 2 shows the change in anxiety preprocedure vs postprocedure by treatment group. Subjects in the lavender group showed a greater reduction in anxiety scores (9.9) compared to the almond oil (5.3) and water (3.6) groups. Table 3 shows the between-group differences for the change in anxiety scores.

**Table 3.** Differences in Anxiety Scores Between Treatment Groups Preprocedure vs Postprocedure

Comparison	Difference Between Groups	95% CI
Lavender vs water	–6.3	–15.4 to 2.8
Lavender vs almond oil	–4.6	–13.5 to 4.3
Almond oil vs water	–1.7	–11.0 to 7.7

None of the differences between groups showed statistical significance at the *P*<0.05 level.

We performed a separate analysis to check for confounding variables. Whether subjects took anxiolytics or analgesics on the day of the scheduled procedure or had the same procedure during the preceding 2 years had no effect on results.

No adverse reactions to lavender or almond oil were reported, but 3 subjects reported that the smell was potent in the lavender group.

## DISCUSSION

This study focused on the anxiolytic and analgesic effects of lavender using 2 questionnaires, the NRS for pain and the STAI for anxiety, in patients scheduled for lumbar epidural injections and lumbar medial branch blocks. Inhalation of lavender resulted in a greater change in anxiety scores after wearing a mask infused with lavender when compared to the almond oil and placebo groups, but this change was not statistically significant. Based on a 2019 systematic review, 54 of 65 randomized controlled trials reported a significant result in favor of lavender use for anxiety when measured by the STAI: a mean change of –5.99 (95% CI –9.39 to –2.59; *P*<0.001).^28^ Inhalation of lavender also resulted in lower pain levels experienced during the scheduled procedure when compared to almond oil and placebo at a clinically relevant although not statistically significant level. Based on studies evaluating clinical meaningfulness for reduction in pain among patients with low back pain, a score of 1.8 to 1.9 on the NRS that was used in our study correlates with a minimum clinically important change.^29^

Different mechanisms have been proposed for the effects of lavender therapy. First is the influence of aroma on the brain through the limbic system via olfaction, and the other is through the direct pharmacologic effects of the essential oil. Lavender contains 2 compounds, linalool and linalyl acetate, both of which have been shown to stimulate the parasympathetic nervous system.^30^ The anxiolytic effects of lavender may be attributable to the antagonizing effects on the N-methyl-D-aspartate receptor and inhibition of the serotonin transporter, which may explain its antiagitation effect in animals.^31^ Another mechanism proposed is the effect of lavender on the serotonin receptor in specific areas of the brain (hippocampus, anterior cingulate cortex, temporal gyrus, insula) through a reduction of its expression and binding ability, the way that a selective serotonin receptor inhibitor medication works.^32^ In animal models, linalyl acetate anxiolytic effects were not observed in anosmic mice, indicating that the effects were triggered by olfactory input. In addition, flumazenil, a benzodiazepine antagonist that competitively inhibits by interacting with benzodiazepine receptor sites on the GABA/benzodiazepine receptor complex, was found to antagonize odor-induced anxiolytic effects, indicating that gamma-aminobutyric acid transmission plays an important role in anxiolytic effects.^33^

Other studies have investigated inhalation of essential oils during interventional spine procedures. Eucalyptus, specifically its constituent 1,8-cineole, was found to decrease anxiety before selective nerve root blocks.^34^ A meta-analysis conducted in 2016 summarized 12 studies that examined the effects of aromatherapy on pain, and a significant positive effect was found in the reduction of pain. Types of aromatherapy included in the studies were lavender, eucalyptus, lemon grass, and rose oil.^35^

The potential for anxiety to alter the course of pain is well known. Evidence shows that the emotional and behavioral responses to pain are strongly guided by 2 related psychosocial factors, fear and anxiety. Individuals who have lower anxiety do not interpret pain as negatively as those with higher anxiety levels, which aids in recovery.^36^ In addition, satisfaction with pain management often has little correlation to pain reduction and is more often associated with communication, staff behavior, and empathy.^37^ A pleasant scent added to a potentially anxiety-provoking experience, such as a fluoroscopically guided medical procedure, may play a role in patient satisfaction.

This study has several limitations. The study administrator was not blinded to which patients received which treatment, and although patients were told they were assigned to 1 of 3 groups and not told which treatment they were receiving, complete blinding, because of the smell of the lavender and almond oil, was not possible. These results are based on a preliminary analysis because of the COVID-19 pandemic that resulted in a temporary pause in all clinical research due to public health guidelines and a shortage of masks that limited the sample size and the duration of the study. Future directions include conducting a larger-scaled study and expanding the procedure pool to include other fluoroscopically guided procedures such as cervical epidurals and cervical medial branch blocks as well as genicular nerve blocks and radiofrequency ablation. As some subjects reported that the smell of lavender was potent, researchers for future studies may consider using a dilution or instilling <5 drops of lavender into a mask. Last, whether instructing patients to breathe slowly and deeply had an effect on outcomes, because all patients were asked to breathe this way, is unknown.

Because of patient tolerance of the intervention, lack of adverse effects, low cost, and clinical effect on pain and anxiety levels, a feasible clinical practice may be to include a lavender scent in the waiting area or preprocedural area.

## CONCLUSION

Lavender may have a clinically meaningful effect on decreasing pain during lumbar epidural injections and medial branch blocks but was not found to have a significant effect on reducing anxiety related to lumbar epidural injections and medial branch blocks.

## Notes

*This article meets the Accreditation Council for Graduate Medical Education and the American Board of Medical Specialties Maintenance of Certification competencies for Patient Care and Medical Knowledge.*
